# Satisfaction with life, depression, anxiety, and stress among adolescent girls in Tehran: a cross sectional study

**DOI:** 10.1186/s12888-022-03757-x

**Published:** 2022-02-11

**Authors:** Sara-Sadat Hoseini-Esfidarjani, Kiarash Tanha, Reza Negarandeh

**Affiliations:** 1 Nursing and Midwifery Care Research Center, Tehran University of Medical Sciences, Nosrat St., Tehran, 1419733171 Iran; 2 Department of Biostatistics, School of Public Health, Iran University of Medical Sciences, ShahidHemmatHighway, Tehran, 1449614535 Iran; 3 Nursing and Midwifery Care Research Center, Tehran University of Medical Sciences, Nosrat St., Tohid Sq., Tehran, 1419733171 Iran

**Keywords:** Satisfaction with life, Depression, Anxiety, Stress, Adolescent girls

## Abstract

**Objective and background:**

Mental health is a widespread field that entails variables such as the presence of positive feelings like satisfaction with life and lack of negative emotions like depression, anxiety, and stress. This research aimed to study the prevalence of depression, anxiety, stress, and satisfaction with life as dimensions of adolescent girls’ mental health in Tehran.

**Methodology:**

The population considered in this cross-sectional study consisted of adolescent girls in the last grade of high school in Tehran. Research samples were selected using multi-stage sampling. The sample size in this study was 491 and the research tools used were standardized questionnaires. Descriptive and inferential statistics included Spearman, ANOVA, and regression tests were used.

**Findings:**

More than half of the adolescents experienced common symptoms of depression, anxiety, and stress. About 30% of adolescents were dissatisfied with life to some extent. Satisfaction with life was negatively correlated with age, depression, anxiety, and stress. Age and depression were predictor variables of life satisfaction based on the regression model.

**Conclusion:**

A considerable percentage of adolescents suffered some form of depression, anxiety, and stress symptoms and were notably dissatisfied with life. Regarding the importance of satisfaction with life in having a joyful life and its role in initiating depression, anxiety, and stress, our findings highlight the need for interventions to prevent depression, anxiety, and stress and enhance life satisfaction among adolescents.

## Introduction

According to the World Health Organization (WHO) definition, “mental health is defined as a state of well-being in which every individual realizes his or her potential, can cope with the normal stresses of life, can work productively and fruitfully, and is able to make a contribution to his or her community” [1]. Since mental well-being is measured using variables such as; satisfaction with life, anxiety, depression, stress, and disappointment, it can be said that these variables are dimensions of mental-health and well-being [2].

Adolescence (10-19 years old) is the period of transition from childhood to adulthood [3] and it is also a critical step that involves the promotion and preservation of important emotional habits that foster mental well-being [4]. Poor mental health can have significant effects on the health and development of adolescents and is associated with various adverse social consequences (that is, alcohol and drug abuse, delinquent behavior, school dropout) [5].

Satisfaction with life is a positive dimension of well-being, which refers to a comparative process in which people assess their quality of life based on their own standards [6]. Regarding satisfaction with life, Moksnes and Espnes showed that the mean score of satisfaction with life among Norwegian girls was 22.31 ± 6.01, and based on the satisfaction with life scale used in this study; girls were a little satisfied [7]. In the study by Al Khatib (2013), the mean score of satisfaction with life among the United Arab Emirates (UAE) students was higher than the neutral level [8]. Moreover, satisfaction with life in adolescents predicts the most salient social (relationship with parents), behavioral (delinquency) [9], and psychological dimensions (depression and anxiety) [10].

In the early years of childhood, diagnosed mental health disorders are higher in boys than girls. However, depressive disorders and anxiety are often more deleterious to girls by adolescence due to inequalities. Because there is usually no intervention during adolescence, these disorders can persist into adulthood and have debilitating long-term consequences for mental health [11]. Young women are also vulnerable to several factors, including poverty-related hardship, physical abuse, less access to education and employment opportunities, and limited mobility [12].

Negative feelings like depression, anxiety, and stress are among the most common mental health problems [13]. According to the American Psychiatric Association and the DMS-5, “Depression is a common and serious medical illness that negatively affects how you feel, the way you think and how you act” [14]. Depression has been known as the first mental health priority among adolescents due to its high prevalence, recurrence, and ability to cause significant impairment [15].. On the other hand, stress is a challenge or threat to well-being. It is a process in which environmental demands exceed an organism’s adaptation capacity, lead to mental and biological changes, and expose people to risks. Stress is an essential part of life because its persistence may lead to various psychological problems such as involvement in high-risk behaviors [16]. In comparison, anxiety is a natural reaction to stress. It can be a beneficial implication that often alerts us in dangerous situations, thereby increasing our readiness to act promptly. Nevertheless, anxiety disorders are different from feelings of nervousness or anxiousness and entail extreme fear or anxiety [17]. According to the WHO report, 10-20% of adolescents throughout the world experience mental health disorders [4]. To date, within published literature, various studies have reported a notable proportion of adolescents experiencing one or more mental disorders. For instance: a systematic review by Polanczyket et al. (2015) showed that the prevalence of mental disorders in children and adolescents in 27 countries was 13.4%.The prevalence of anxiety disorder was 6.5% and depressive disorder was 2.6% [18]. Sandal et.al (2017) study among students of grades 9 to 12 attending public schools in India showed that 65.53% had depression (in mild-extremely severe forms), 80.85% suffered from anxiety (in mild-extremely severe forms), and 4.02% had stress (mild-extremely severe) [19]. Noteworthy, few studies have been conducted in this field and population in Iran. However, we wish to note that such problems are high in Iran**.** A meta-analysis partly evidences this by Sajjadi et al. (2013). Based on Sajjadi’s study, the prevalence of depression among Iranian adolescents was 13.5 to 43.5%. This was, however, revealed using different tools. One of the most important factors that has been implicated among adolescents experiencing depression is female gender and in spite of the importance of depression in adolescence, many other areas deserve attention [20].

Since adolescents with unsuitable mental health conditions are exposed to social exclusion, educational problems, and physical illnesses [4], paying attention to the mental health of this group is a priority for health promotion programs in the country. Given the sensitivity of this group, and since no extensive information was found on this subject and population in Iran, the present study has been conducted to measure satisfaction with life, depression, anxiety, and stress as the dimensions of mental health in a representative sample of adolescent girls in Tehran. Evidence from this study can be a basis for estimating the extent of adolescents’ mental health in the country.

## Methodology

This was a cross-sectional descriptive-analytical study conducted in February 2018 till March 2018 in Tehran, capital of Iran. The study population was adolescent girls in the last grade of high school. The inclusion criteria included: being female, being in the final grade of high school, residing in Tehran. The exclusion criterion had a physical disability.

The sample size was estimated for the Infinite population by using the following formula:

*n* = Zα/22 *p*(1-p) / d2 where, Zα/2 is 1.96, d or the margin of error is 0.05, and p is a prevalence of depression which is 30% on the Montazeri et al. study [21],(as well cluster sampling effect of 1.5 was considered. Then by adding Five percent for non-responses and incomplete questionnaires, 508 questionnaires were distributed. The participants were selected using a multi-stage sampling method. Nineteen educational districts of Tehran were first placed in 4 clusters (such that the first cluster included districts; 15, 16, 17, 18, 19; second cluster were districts; 9, 10, 11, 12, 13, 14; the third cluster included districts; 6, 7, 8, 5, 2; and the fourth cluster were districts; 1, 3, 4). Then, one district was selected from each cluster randomly (in total, four districts were selected from 4 clusters). The four selected districts were districts number 1, 5, 10, and 15. Then, a public school and a non-public school were selected from each of the four selected districts. In the last step, simple random sampling was used to select students from the school. Since all subjects were under 18 years old, after arrangement with the principals of schools, consent forms were given to the student to be signed by their parents. Parents who consented that their children should participate in the research signed the form and returned it. The goals and general information about the study were explained to the students, it was voluntary to participate, and anonymity of data was maintained at all times.

Demographic information like age, father’s job, and mother’s job were asked. Alongside, two data gathering tools were used in this study. Satisfaction with life scale (SWLS) was used to measure satisfaction with life. The tool has five items scored on a 7-point Likert scale from strongly agree to strongly disagree. The range of scores was 5-35, and the higher scores showed higher satisfaction. In this classification, those who obtained 31 to 35 were extremely satisfied, 26 to 30 satisfied, 21 to 25 slightly satisfied, 20 neutral, 15 to 19 slightly dissatisfied, 10-14 dissatisfied, and 5-9 extremely dissatisfied. Considering that Cronbach alpha and Inter-class Correlation Coefficient (ICC) are common metrics for reliability, Bayani et al. (2007) obtained the reliability of satisfaction with life scale among law students using Cronbach alpha and ICC of 0.83 and 0.69, respectively [22]. The Cronbach alpha in this study on the adolescent girls of Tehran was 0.90, and Inter-class ICC was 0.82.

Depression, anxiety, and stress were measured using the Depression, Anxiety, Stress Scale-21 (DASS-21) questionnaire. The Depression, Anxiety and Stress Scale - 21 Items (DASS-21) is a set of three self-report scales designed to measure the emotional states of depression, anxiety, and stress. The tool has 21 items scored on a 4-point Likert scale (that is, never to almost always). Seven questions (questions 3, 5, 10, 13, 16, 17, 21) of this scale are related to depression, seven questions [2, 4, 7, 9, 10, 15, 19] assess anxiety and seven questions (1, 6, 8, 11, 12, 14, 18) assess stress. On the depression subscale, the scores were classified as follows: 0-9 normal condition, 10-13 mild depression, 14-20 moderate depression, 21-27 severe depression, and above 28 as extremely severe depression. The scores on the anxiety subscale were: 0-7 normal condition, 8-9 mild anxiety, 10-14 moderate anxiety, 15-19 severe anxiety, and above 20 was extremely severe anxiety. On the stress subscale, the scores were: 0-14 normal condition, 15-18 mild stress, 19 to 25 moderate stress, 26-3 severe stress, and above 33 extremely severe stress. In this study, the Persian version of the DASS-21 questionnaire, which has been translated and validated into Persian by Sahebi et al. (2005) was used [23]. Najafi-Kalyani et al. (2010), in their study among associate, bachelor, and Ph.D. students, calculated the validity of this questionnaire by using Cronbach alpha for stress, anxiety, and depression was 0.80, 0.86, and 0.83, respectively [24]. The Cronbach alpha of the whole DASS-21 questionnaire in the present study was 0.87, and ICC was 0.77. Also, the Cronbach alpha of the DASS-21 questionnaire subscales of depression, anxiety, and stress in the present study was 0.83, 0.78, and 0.80, respectively; the ICC for subscales of depression, anxiety, and stress was 0.85, 0.77, 0.85, respectively.

Frequency and percentage were used to describe categorical variables, and in the case of continuous variables, mean and standard deviation were used. The Spearman’s test was used for assessing bivariate correlation. Multiple regression models were performed to determine factors related to satisfaction with life using stepwise methods. *Considering* the design *effect in cluster* sampling, all analysis was performed in a survey using Stata 13 (*StataCorp*. 2013. *Stata* Statistical Software: Release *13*. College Station, TX: *StataCorp* LP). The significant level was considered of *P* < 0.05.

## Results

In this study, from 508 distributed questionnaires, data from 491 questionnaires were analyzed, yielding a response rate of 96.7%. The mean age of adolescents was 17.23 ± 0.77, with a range of 13-20. Most fathers were employed (80.8%), and only 2.9% were unemployed. The rest were retired or died. The majority of mothers were housekeepers (77.7%), and 20.8% were employed. The mean and standard deviation of depression, anxiety, and stress subscales and their frequency distribution based on severity are shown in Table 1. Accordingly, More than half of the students showed some degree of depression, anxiety, and stress. The status of satisfaction with life, based on cut point, its mean scores, and the standard deviation, is presented in Table 2. In total, a notable percent of adolescents experienced severe and extremely severe forms of depression (16.8%), anxiety (28.3%), and stress symptoms (19.1%). About 30% of them had some degree of dissatisfaction with life.

**Table 1:** Frequency distribution of depression, anxiety and stress in adolescent girls in Tehran (*n* = 491)

No.	Severity	Depression			Anxiety			Stress		
		Number	Percent	95% CI	Number	Percent	95% CI	Number	Percent	95% CI
1	Normal	232	48.34	42.83, 53.90	198	41.72	34.14, 49.71	232	47.68	42.92, 52.48
2	Mild	74	15.01	11.81, 18.90	37	8.17	6.07, 10.91	87	18.98	17.37, 20.71
3	Moderate	95	20.53	17.05, 24.51	105	22.52	17.96, 27.83	72	15.45	11.41, 20.60
4	Severe	35	7.28	4.34, 11.99	51	10.60	8.24, 13.53	57	11.92	8.79, 15.97
5	Extremely Severe	43	8.83	6.11, 12.60	80	17.00	13.57, 21.08	27	5.96	3.52, 9.91
	Mean (SD)	11.56 (0.35)		10.74, 12.39	10.73 (0.57)		9.38, 12.08	16.11 (0.51)		14.89, 17.33

**Table 2:** Frequency distribution of satisfaction with life in adolescents girls of Tehran (*n* = 491)

Satisfaction with life	Extremely satisfied	Satisfied	slightly satisfied	neutral	slightly dissatisfied	Dissatisfied	Extremely dissatisfied
Number	79	137	116	13	63	51	27
Percent	16.78	27.59	23.84	2.87	12.36	11.26	5.30
95% CI	11.67	22.64	16.84	1.90	9.96	8.54	3.72
	23.52	33.17	32.61	4.31	15.25	14.70	7.50
Mean (SD)	23.12 (0.50), 95% CI: 21.94, 24.30						

The result of the Spearman’s test showed that there is a correlation between satisfaction with life and anxiety (*r* = − 0.364, *p* < 0.001), depression (*r* = 0.588, *p* < 0.001), stress (*r* = − 0.401, *p* < 0.001) and age (*r* = − 0.155, *p* < 0.001).

A regression model was fitted for the four variables (depression, anxiety, stress, and age). Since anxiety and stress were not significant, by eliminating them, the regression coefficient and *p*-value were changed (Table 3). The regression analysis results indicated that depression and age have a significant association with satisfaction with life after adjustment for anxiety and stress.

**Table 3:** the result of multiple regression analysis between depression and age as predictor variables and satisfaction with life as a criterion

variables	Beta	Standard deviation	*p*-value
Depression	- 0.432	0.023	< 0.001
Age	−1.62	0.239	< 0.001

## Discussion

In this study, based on the DASS-21 questionnaire, more than half of adolescents showed a notable degree of depression, anxiety, and stress. Specifically, our study identified that a considerable proportion of adolescents experienced severe and extremely severe forms of depression (16.8%), anxiety (28.3%), and stress (19.1%). Elsewhere, such remarkably high proportions of the mental problems under consideration in our study have been reported. For instance the results of Kordi et al. (2015) study among the female high-school students of Mashhad showed that 21.7% experienced severe and extremely severe self-reported depressive symptoms, 24.3% had severe and extremely severe self-reported anxiety symptoms and 21.8% had severe and extremely severe self-reported stress symptoms [25]. The systematic review by Montazeri et al. (2013) in Iran showed that the prevalence of depression among the different populations of Iran was reported as 5.69-73%. The highest amount was related to female high-school students in Firozkogh city [21]. According to Sahoo and Khess’s (2010) study, the depression, anxiety, and stress symptoms were 18.5, 24.4, and 20%, respectively, in 17-22 years old students in India [26]. Generally, the above-noted findings from previous studies [18–20] are consistent with the present study results. The systematic review study of Polanczyk et al. (2015) estimated that about 241 million youths worldwide suffer from some form of mental disorder [18]. Depressive disorders and anxiety disorders are highly prevalent in the population. At a global level, over 300 million people are estimated to suffer from depression, equivalent to 4.4% of the world’s population. The number of persons with common mental disorders globally is going up, particularly in lower-income countries, because the population is growing and more people are living to the age when depression and anxiety most commonly occur [27]. Regarding the depression, anxiety, and stress in female students in the present study and since the continuance of mental health problems have detrimental effects on academic achievement as well as on the potential that adolescent girls might have. We recommend that interventional studies in this context could be beneficial to avert such notable levels of mental health problems revealed in this study.

This study indicated that about 30% of adolescents were dissatisfied with life dissatisfaction. The results of Okwrji’s (2016) study using SWLS showed that among adolescents aged between 16 and 19 years, 32.3% were dissatisfied with life [28].. This is consistent with the findings of this study. In a 4-year longitudinal study in Hong Kong, Shek showed that the mean satisfaction with life, based on SWLS, reduced from 19.38 ± 5.37 to 18.54 ± 5.27. Generally, based on this tool, satisfaction with life among adolescents was lower than the neutral level. This means they were slightly dissatisfied with life [29]. This is partly attributed to the fact that during adolescence, the sensitivities increase, and the goals renew. Thus, satisfaction with life in this period is an important psychological variable. Through developing cognitive capabilities, the adolescent can evaluate and predict the primary needs with higher precision [30]. Based on the findings of this study, satisfaction with life in a considerable percent of adolescents was below moderate.

This study showed that satisfaction with life is correlated with depression, anxiety, and stress. According to the regression model, depression and age are correlated variables of satisfaction with life. It seems that performing interventions to moderate and change life dissatisfaction is necessary. We hope that the results from our study that highlighted the correlation between various mental problems and satisfaction with life could guide such interventions. Similarly, the previous studies showed that lower satisfaction with life among adolescents and adults predicts higher depression levels [28, 31]. Shi (2015), using SWLS among 2925 students in China, showed that stress has a negative correlation with satisfaction with life (r = − 035; *p* < 0.01) [32]. Guney et al. (2010) study in Turkey showed that in 364 students in the age range of 19 to 25 years old, the satisfaction with life, that was measured with SWLS, has a negative correlation with anxiety which is measured by Beck Anxiety Inventory (r = − 0.26, p < 0.01) [2].

The results of Moksens & Espnes were consistent with the results of this study regarding the relationship of age and satisfaction with life among male Norwegian adolescents [7]. Contrary, the Al-Attiyh study showed that age has no significant association with satisfaction with life [33]. Aging comes with biological and mental changes that can affect people’s goals and wishes in life and their feelings about satisfaction with life. Aging is also associated with the changes in the role of individuals and increasing responsibilities. These changes may increase or decrease the level of satisfaction with life, based on an individual’s perception of her/his conditions.

One of the strengths of this research was that it used a representative sample size. On the other hand, this study is not without limitations. The measures used for screening are not considered diagnostic tools but rather identify those who may need further evaluation and possible intervention. A specialist should make a diagnosis with specialized clinical examinations. Also, the study only considered adolescent girls in their final grade. Owing to the differences in life experiences of girls and boys, these findings could only be generalizable in the context of adolescent girls.

## Conclusion

The results of this study indicated that a significant proportion of adolescents experience some form of depression, anxiety, and stress and experience sub-optimal satisfaction with life. Findings also suggest that depression and age have an impact on life satisfaction. Therefore, appropriate screening programs and interventions are essential to identify and treat depression in adolescents.
